# Strawberry soluble solids QTL with inverse effects on yield

**DOI:** 10.1093/hr/uhad271

**Published:** 2023-12-21

**Authors:** Zhen Fan, Sujeet Verma, Hana Lee, Yoon Jeong Jang, Yu Wang, Seonghee Lee, Vance M Whitaker

**Affiliations:** IFAS Gulf Coast Research and Education Center, Horticultural Sciences Department, University of Florida, Wimauma, Florida 33597, USA; IFAS Gulf Coast Research and Education Center, Horticultural Sciences Department, University of Florida, Wimauma, Florida 33597, USA; Department of Food Science and Human Nutrition, IFAS Citrus Research and Education Center, University of Florida, Lake Alfred, Florida 33850, USA; IFAS Gulf Coast Research and Education Center, Horticultural Sciences Department, University of Florida, Wimauma, Florida 33597, USA; Department of Food Science and Human Nutrition, IFAS Citrus Research and Education Center, University of Florida, Lake Alfred, Florida 33850, USA; IFAS Gulf Coast Research and Education Center, Horticultural Sciences Department, University of Florida, Wimauma, Florida 33597, USA; IFAS Gulf Coast Research and Education Center, Horticultural Sciences Department, University of Florida, Wimauma, Florida 33597, USA

## Abstract

Sugars are the main drivers of strawberry sweetness, and understanding their genetic control is of critical importance for breeding. Large-scale genome-wide association studies were performed in two populations totaling 3399 individuals evaluated for soluble solids content (SSC) and fruit yield. Two stable quantitative trait loci (QTL) on chromosome 3B and 6A for SSC were identified. Favorable haplotypes at both QTL for SSC decreased yield, though optimal allelic combinations were identified with reduced impacts on yield. Metabolites in the starch and sucrose metabolism pathway were characterized and quantified for 23 contrasting genotypes in leaves, white fruit, and red fruit. Variations in sucrose concentrations/efflux indicated genetic variation underlying sucrose accumulation and transportation during fruit ripening. Integration of genome-wide association studies and expression quantitative locus mapping identified starch synthase 4 (FxaC_10g00830) and sugar transporter 2-like candidate genes (FxaC_21g51570) within the respective QTL intervals. These results will enable immediate applications in genomics-assisted breeding for flavor and further study of candidate genes underlying genetic variation of sugar accumulation in strawberry fruit.

## Introduction

Strawberries (*Fragaria × ananassa,* 2*n =* 8*x =* 56) are among the most economically important fruit crops globally and are widely appreciated for their unique aroma, flavor, and nutritional value. A critical determinant of strawberry flavor and consumer preference is fruit sugar content, which highly influences sweetness perception and consumer preference [1]. Although volatiles can enhance sweetness and liking to some extent, sugars, including glucose and sucrose, are major contributors to sweetness perception, with >0.6 correlations between soluble solids and sweetness intensity and between total sugars and sweetness intensity in large sensory-chemical studies [1, 2]. As the global demand for sweeter, more flavorful strawberries continues to rise, understanding the genetic underpinnings of sugar accumulation in strawberries has become critical for breeding programs.

Several studies have identified quantitative trait loci (QTL) linked to soluble solids content (SSC) in strawberry [3–8]. Many of these studies utilized segregating populations derived from a limited number of crosses and relied on low-resolution genetic maps for QTL detection, limiting their utility. Vallarino et al. examined QTL for specific metabolites that collectively contribute to SSC [9]. Although QTL were detected for individual sugars, organic, and amino acids, only 13% of QTL were repeatable across years, emphasizing impacts of environments on primary metabolites. In Michigan and Oregon populations, one SSC QTL with moderate effect was identified on linkage group 6A [10]. Collectively, these studies underscore the importance of detecting QTL across multiple environments and populations in order to find suitable and repeatable marker-trait associations for target breeding programs. Additionally, despite a negative correlation observed between SSC and yield at the phenotypic and genetic levels in University of Florida strawberry breeding population [11], no QTL with effects on both have been described.

Although SSC is an excellent approximation of total soluble sugars in strawberry fruit, the precise quantification of individual soluble sugars is helpful for understanding sugar accumulation and transportation during fruit ripening [12]. Plants convert light into energy via photosynthesis, producing carbon assimilates from carbon dioxide and water. As in many other plants, sucrose is the main sugar in strawberry that is transported long distances to sink tissues such as fruit [13]. Before storage in the vacuole, these sugars undergo at least three transmembrane translocations through phloem unloading and post-phloem transport [12]. Apoplasmic unloading allows sugar uptake against concentration gradients, but requires sugar transporters on the membrane [12]. Of these, sucrose transporters (SUTs) and the SWEET transporter family [14] are well studied across diverse plant species, and both are abundantly represented in the strawberry genome [15, 16]. Using three varieties, multiple SUTs were found positively correlated with fruit soluble sugar content [17]. Once transferred to the fruit’s parenchyma cells, tonoplast sugar transporters (TSTs) facilitate the movement of soluble sugars into the vacuole for storage. A TST in diploid strawberry (*FvTST1*) was identified and functionally validated in transgenic tomato plants. Transient expression of *FvTST1* in strawberry fruits enhanced both fruit ripening and sugar accumulation [18]. In strawberry fruit, a significant portion of sucrose is hydrolyzed into fructose and glucose, a process catalyzed by invertases present in sink tissues [19]. Several such invertases have been identified in the octoploid strawberry genome, with one upregulated during strawberry development and strongly expressed in ripe fruit [20] [21].

The main goal of this study was to (1) identify and validate QTLs associated with sugar content using multiple, large genome-wide association studies (GWAS) populations phenotyped in multiple environments; (2) identify haplotype combinations with positive effects on SSC and limited loss of yield; (3) examine sugar metabolites across three tissues to gain insights into sugar flux during fruit ripening; (4) and identify candidate genes underlying SSC QTLs with the integration of expression quantitative locus mapping (eQTL) results.

## Results

### Two stable SSC QTL across harvests, years and populations

The SSC and yield data were normally distributed for both the diversity population of cultivars and advanced breeding selections (*n* = 1778) and the multi-family seedling population (*n* = 1621) (Fig. S1). A Pearson’s correlation analysis reaffirmed a negative correlation of r = −0.22, r = −0.16 between SSC and yield for the diversity population and multi-family population, respectively. For the diversity population, the average SSC was 8.1% (SD = 0.81), and the average yield value was 547.4 g (SD = 149.5, 16-week span). For the multi-family set, average SSC was 8.7% (SD = 1.2) and average yield was 192.3 g (SD = 91.4, 11-week early-season span).

**Figure 1:**
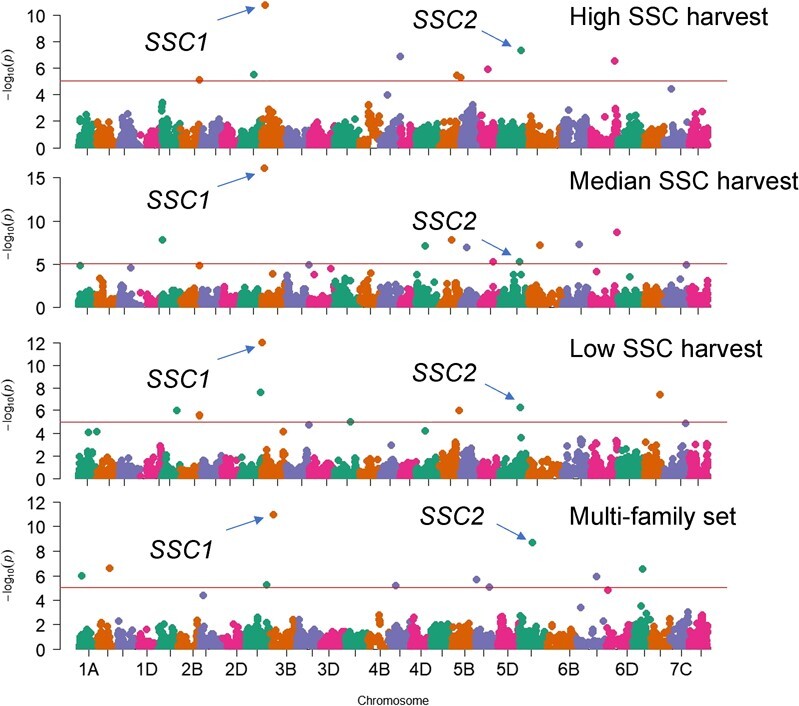
Manhattan plots of GWAS results for three separate SSC measurements for the diversity population and the mean of two SSC measurements for the multi-family population.

The narrow-sense heritabilities were 23.9%, 45.1%, and 31.4% for three SSC measurements (high/median/low SSC harvests), respectively. Across all three harvests, GWAS consistently detected two QTL for SSC on chromosome 3B, *SSC1*, and 6A, *SSC2*, at 3 Mb and 8 Mb, respectively (Figs.1 and2, Supplementary Data S1). The phenotypic variance explained (PVE) by the lead markers ranged from 1.7% to 2.8% for *SSC1* and 1.2% to 2.2% for *SSC2* (Supplementary Data S1)*.* For the multi-family set (*h*^2^ = 28.2%), despite a slight shift in the physical location possibly due to the family structure, the QTL on chr 3B and 6A were the most significant (*P* = 1.15E-11 and 2.29E-9, respectively) (Fig. 1).

**Figure 2:**
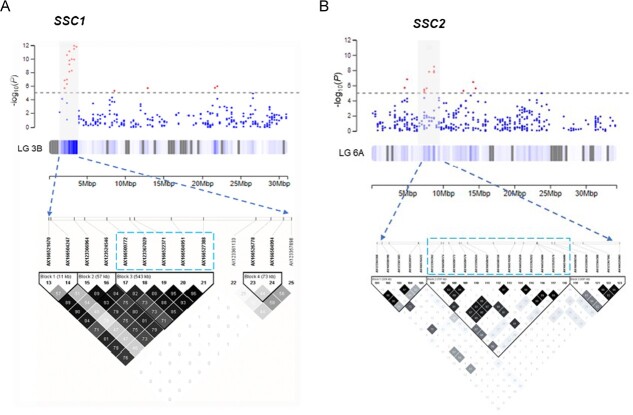
At top are GWAS results of marker significance for the *SSC1* (A) and *SSC2* (B) loci on chromosomes 3B and 6A, respectively. At bottom are linkage blocks in the QTL region. Darker color represents higher linkage disequilibrium (R^2^). Markers highlighted in blue boxes were used for haplotype analyses.

### Haplotype analyses revealed inverse allele effects on yield and sugar content

**Figure 3:**
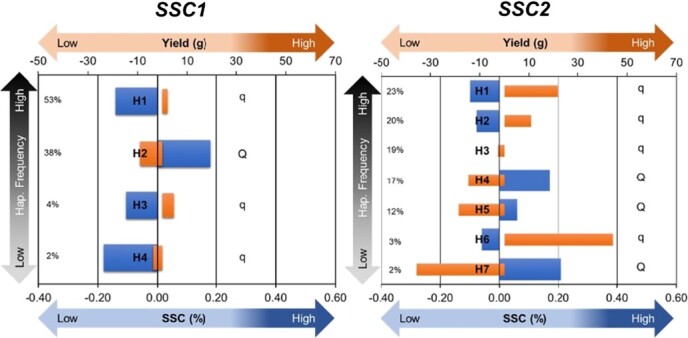
Haplotype effects of *SSC1* and *SSC2* on yield (orange color) and soluble solids content (SSC, blue color). Haplotype frequencies for individual haplotypes are annotated on the left sides of the plots. The *x* axes represent deviations from the population means.

**Figure 4:**
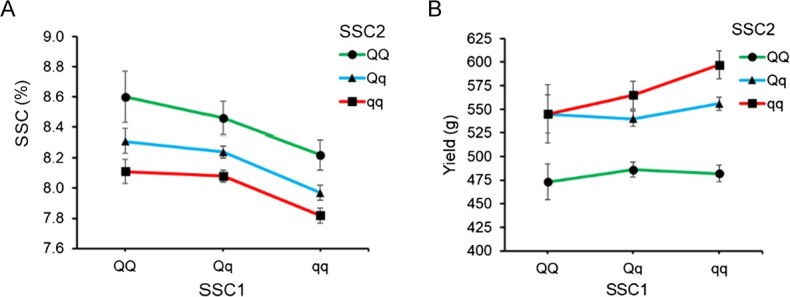
Soluble solids content (A) and yield (B) for QTL genotypes at *SSC1* and *SSC2*, as determined by haplotype combinations, in a diversity population of nearly 1800 individuals.

Haploblocks were determined for *SSC1* and *SSC2* (Fig. 2, Table S1): four haplotypes at chr 3B and seven haplotypes at chr 6A represented >98% of the genetic diversity at each QTL. Haplotype effects for SSC and yield were in opposite directions for each haplotype, except H4 on chr 3B, which was least frequent (Fig. 3). Markers within *SSC1* QTL haploblock were in higher LD as compared to *SSC2* QTL markers (Fig. 2). At both QTL, haplotypes associated with positive effects on SSC were labelled as “Q”, and haplotypes associated with negative effects on SSC were labelled as “q” (Fig. 3, Table S1). An additive impact of QTL genotypes without significant interaction between the two loci (*P* > 0.05) was observed, along with a consistently inverse relationship between SSC and yield (Fig. 4). Together, the allele combinations at the two loci explained ranges of 0.8% in SSC and 124 g in marketable yield in the diversity set (Table 1). Breeding possibilities for achieving a balanced level of SSC and yield in strawberries depend on the QTL source and the Q/q dosages. Generally, the Q haplotypes on *SSC1* had smaller negative effects on yield than *SSC2* (Table S1)*.* The top three haplotype combinations that substantially increased SSC and minimized yield penalty were 1Q:1Q, 2Q:0Q, and 2Q:1Q (Table 1).

**Table 1:** Dosage effects of two SSC QTL in the diversity population. Values of SSC and yield are deviations from the population mean

Order of Q dosage	Q Dosage	SSC (%)	Yield (g/season)
SSC1:SSC2 (0:0)	0	−0.29	43.79
SSC1:SSC2 (1:0)	1	−0.03	11.56
SSC1:SSC2 (0:1)	1	−0.13	3.17
SSC1:SSC2 (1:1)	2	0.14	−13.10
SSC1:SSC2 (2:0)	2	0.03	−7.61
SSC1:SSC2 (0:2)	2	0.18	−71.05
SSC1:SSC2 (1:2)	3	0.35	−67.30
SSC1:SSC2 (2:1)	3	0.23	−7.60
SSC1:SSC2 (2:2)	4	0.49	−80.12

### Sugar flux during late ripening

**Figure 5:**
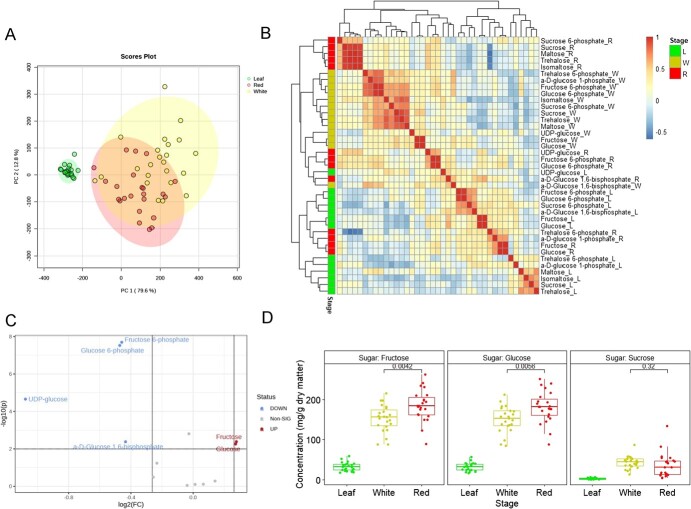
(A) PCA score plot based on sugar metabolites. Dots and eclipses are colored according to tissue types. (B) Heat map of Pearson’s correlations among sugar metabolites measured for different tissue types (L: leaf, W: white fruit, R: red fruit). (C) Volcano plot shows –log *P*-values on the *y* axis and log2 of fold change on the *x* axis, comparing sugar metabolites of red fruits against white fruits. Upregulated metabolites are in red, and downregulated are in blue. (D) Concentrations of three sugars in different tissue types. *P*-values from Student *t*-test between white and red samples were also plotted.

Using abundances of 13 sugar metabolites in the starch and sucrose metabolism pathway (Fig. S2, Supplementary Data S2), samples from leaves and fruits were clearly separated by PC1 (Fig. 5A). Despite some overlap, the majority of white and red fruit samples were separated by PC2. Correlations among metabolites revealed a strong link between fructose and glucose (mean r = 0.99), fructose 6-phosphate and glucose 6-phosphate (mean r = 0.97) and among sucrose, maltose, trehalose, and isomaltose within each tissue type (Fig. 5B). Generally weak correlations were found among sugar metabolites across different tissues (Fig. S3), but abundances of fructose (r = 0.34) and glucose (r = 0.27) in leaves and red fruits were correlated. During the late ripening stage in fruits changing color from white to red, a rise of fructose and glucose was accompanied by a decline of UDP-glucose, glucose 6-phosphate, fructose 6-phosphate, and αD-glucose 1,6-bisphosphate (Fig. 5C). In that short time window, fructose increased from 152.0 to 185.0 mg/g dry matter and glucose increased from 150.0 to 181.0 mg/g dry matter (Fig. 5D). Although on average no significant change of sucrose was observed between white and red fruits, a large genetic variation for sucrose efflux (the difference of sucrose concentrations between white and red fruits) was found in this transition (coefficient of variation (CV) = 4.50), as well as in all three tissue types (CV = 0.73, 0.36, 0.83), suggesting genetic variation underpinning sucrose transportation and accumulation. The high-sugar cultivar “Florida Beauty” [21] accumulated 86.3 mg/g more sucrose in red stage than white stage, the highest among all tested individuals.

### Candidate genes underlying *SSC1* and *SSC2*

**Figure 6:**
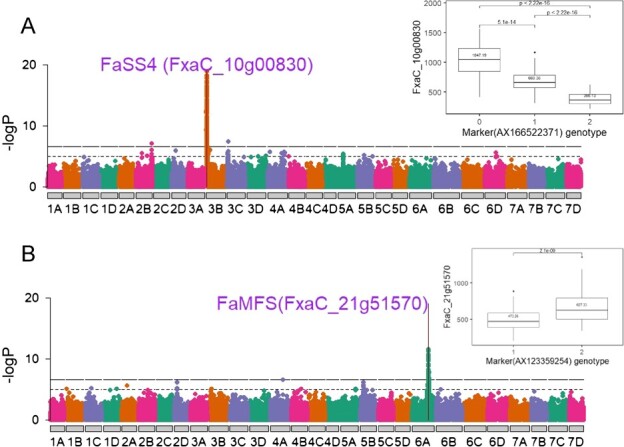
Manhattan plots of *FaSS4* (FxaC_10g00830) and *FaMFS* (FxaC_21g51570) gene expression GWAS. Marker dosage effect on gene expression is plotted on the right panel. Shared markers between eQTL and SSC GWAS peaks were chosen.

Integration of GWAS and expression QTL analyses allowed identification of candidate genes underlying both SSC QTL. Three markers (AX-166522371, AX-166527388, and AX-166504951) inside the *SSC1* were used for candidate searches. Among seven genes sharing cis-eQTL with *SSC1*, BLAST indicated that FxaC_10g00830 was similar to *starch synthase 4* (*SS4*). A maximum-likelihood (ML) phylogenetic tree using starch synthase genes in *Zea mays* and *Arabidopsis thaliana* confirmed the annotation (Fig. S4). An additive effect on gene expression was observed using a co-segregating marker (Fig. 6A). A total of 46 genes shared cis-eQTL with the *SSC2* GWAS signals (AX-123359254, AX123525576, AX-123362922, and AX-166525659), which was evident from low LD among *SSC2* markers. Among them, the homolog of FxaC_21g51570 in *Fragaria vesca* was annotated as a *major facilitator superfamily* protein (*MFS*). BLAST searching its sequence to UniProt database revealed that it contained the transmembrane domain required for a sucrose transporter protein (SUT) [22]. An ML phylogenetic tree including a comprehensive list of grass SUTs [23] placed FxaC_21g51570 sister to *AtSUC3* (Alias, *AtSUT2*) within group 3 despite a large phylogenetic distance (Fig. S5). Similarly, an additive effect on gene expression was observed using a co-segregating marker (Fig. 6B), though no homozygotes for the low-expression allele were sampled due to their rare occurrence.

## Discussion

Environmental factors usually exert a significant influence on primary metabolites such as sugars in fruit [1], due to their involvement in complex biochemical pathways. In both GWAS populations studied, the heritability of SSC ranged from a low to moderate 23.9% to 45.1%, consistent with prior estimates [24, 25]. Despite this reduced genetic variation in some years/populations, large-scale GWAS allowed identification of two stable SSC QTL. Despite only ~2% of phenotypic variation explained by each QTL, four doses of favorable alleles increased SSC by 0.8%, compared to zero doses. Based on a previously developed sweetness and SSC regression model, a rise of 0.8% provides a substantial difference in sweetness perception [1]. Either one or both of those QTL appear to be located in the same chromosome groups/linkage groups as previously reported SSC QTL [4–6]. However, given that prior research primarily employed markers such as AFLP, SSR, STS, and SCAR [6, 26], we are not able to verify whether they are the same QTL. Nevertheless, the apparently recurring identification of SSC QTL across studies suggests that allelic diversity of *SSC1* and *SSC2* are preserved in multiple breeding programs across the globe.

While some studies report an inverse relationship between yield and SSC [4, 8, 25], others find no such link [5, 8]. This discrepancy among studies can likely be attributed to different environments and/or distinct genetic backgrounds of the parents, as all aforementioned mapping studies were established based on a single or few biparental populations. In the UF breeding population, an inverse additive genetic relationship between SSC and yield was evident in an earlier study [11]. In the present study using large GWAS populations, it has become apparent that this trade-off is largely explained by two QTL, both strongly exhibiting this inverse relationship. The trade-off could be attributed to either linkage drag or pleiotropic effects—scenarios both commonly observed in domesticated crops. For instance, in tomato, a recombinant line successfully decoupled a linkage drag that previously contributed to increased disease resistance at the cost of reduced fruit size [27]. Another case was observed in *sucr* (sucrose accumulator gene) introgressed tomato lines, in which increased sucrose was accompanied by reduced ripe fruit weight and seed set accumulation [28]. In the case of sugars and yield in strawberry, no cases of linkage drag have yet been observed, suggesting pleiotropic effects for *SSC1* and *SSC2* due to the basic physiological tradeoff between fruit load and fruit sugar content.

During strawberry ripening, soluble sugar and organic acids accumulate, while free amino acids are reduced [29]. Our work focused on sugar metabolites in the starch and sucrose metabolism pathway pivotal to the organoleptic characteristics of strawberry fruits. High correlations among sugar metabolites, such as the cluster of sucrose, maltose, trehalose, and isomaltose and of fructose and glucose across tissues were consistent with previous metabolite profiling in fruit [9]. Since sucrose is the main phloem loading substrate for strawberry [13], the slight decline of sucrose coupled with large increase of fructose and glucose during the last ripening period indicates high invertase activity in the receptacle. Therefore, contrary to tomato [30], genetic diversity of invertase was limited in our breeding population. On the contrary, a high genetic variation of sucrose efflux during late ripening stage was observed among sampled genotypes, indicating allelic diversity underlying pathways involved in sucrose loading/unloading. In some fruit species, sucrose can also be synthesized in the cytosol from UDP-glucose and fructose-6-phosphate by sucrose-phosphate synthase (SPS) and sucrose-phosphate phosphatase (SPP) or from fructose and UDP-glucose via sucrose synthase (SuSy) [12]. No significant correlation between fructose and sucrose was observed in any tissue types, but a high positive correlation of 0.72 between fructose-6-phosphate and sucrose was observed in white fruit, hinting at the active role of SPS during fruit ripening.

Higher sugar content in fruit of some genotypes could reflect increased assimilation from plant photosynthetic tissue or an increased fruit sink strength. Although photosynthetically fixed carbon is initially allocated to sucrose, the overflow exceeding sucrose storage capacity is converted to starch in source strawberry leaves [31]. Consequently, genes modulating the efficiency of starch synthesis and its subsequent degradation can impact the sugar content of sink fruits. *Starch synthase 4* (*SS4*) is involved in the initiation of the starch granule and controls diel turnover rate [32–34]. Overexpression of *SS4* increases starch accumulation in *Arabidopsis* leaves, as well as sink organs such as potato tuber [35]. Therefore, higher expression of *SS4* in the *SSC1* QTL region may lead to an increase of sugar content in sink fruits, although future work is required to validate its biological mechanism. Many enzymes coordinate the process of sugar partitioning and long-distance translocation. Among them, sugar transporters utilize the proton motive force across the plasma membrane to actively load/unload sucrose against its concentration, a key step in apoplasmic loading/unloading [14]. The candidate gene FxaC_21g51570 in the *SSC2* region contains a transmembrane transporter domain. A gene tree built with grass SUTs placed FxaC_21g51570 in group 3 (SUT2), which is highly expressed in sink tissues across several species [23]. Paralogs of SUT2 in fruits like peach and apple have shown a strong correlation with sucrose accumulation [36, 37].

In conclusion, sugars in strawberry fruit are the main drivers of consumer liking. However, a trade-off between fruit sugar content and yield was previously shown [11] and confirmed here. To dissect that relationship, large-scale GWAS were conducted. Two stable QTL were identified for SSC, with strong and inverse effects on yield for each. Therefore, optimal allele dosage combinations were determined that enhance sweetness while minimizing impacts on yield. Based on metabolite profiling and eQTL, candidate genes at both sugar QTL were identified that appear to be involved in sucrose accumulation and transportation during fruit ripening. These results enable immediate applications in genomics-assisted breeding for flavor and suggest novel hypotheses for sugar accumulation in strawberry fruit.

## Materials and methods

### Genome-wide association study populations

Two University of Florida strawberry breeding populations were independently analyzed. A diversity population included advanced breeding selections spanning five seasons from 2016 to 2021, totaling 1778 individuals. These breeding selections represented the diversity present across the breeding program during this period. Each year, between 411 (2016–17 season) and 452 (2017–18 season) genotypes were planted, and between 67 to 140 common genotypes were replicated across consecutive years. The pedigree of each individual was confirmed using marker data. A multi-family seedling population was composed of unselected seedlings generated in 4 years totaling 1621 individuals. They were distributed in much larger full-sib families (*n* = 25) compared to the diversity set, with full-sib family sizes ranging from a maximum of 77 to a minimum of 29 individuals. The families in each year represented a partial diallel mating design with connectedness of parents both within and across years. Parentage checking and pedigree confirmation were performed using SNP markers for every individual.

The experimental field design of both diversity and multi-family populations followed randomized complete block designs with five replicates for the diversity set and three replicates for the multi-family set, with one runner plant per replicate. For the diversity set, SSC was measured five times during each season with a handheld refractometer, and yield was measured as total marketable yield in a 16-week span from late November to early March. For the multi-family population, SSC was measured twice, and yield was measured in an 11-week span. SSC data of the diversity population for the seasons 2016–17 and 2017–18 overlapped with a previous genomic selection validation study in which further details of methods are provided [38]. Yield data from the diversity population were also utilized in a previous study [39].

### Genotypic data

DNA was extracted from young leaf tissues and submitted to Affymetrix for Axiom™ SNP array genotyping. All individuals were genotyped with one of the three Affymetrix Axiom™ SNP arrays: IStraw90 [40], IStraw35 [41], and 50 K FanaSNP [42]. A common set of 5264 polymorphic SNP markers with consistent calls across all three Axiom™ arrays was selected for GWAS and subsequent genetic analyses.

### Genome-wide association study and haplotype analysis

For the diversity set, in each year the harvests with the average high, median, and low SSC out of the five harvests in each season were chosen for further analysis across years. The high, median, and low SSC harvests were determined according to the average SSC value across the whole population at that harvest date. For example, the harvest date with the highest average was assigned as high SSC harvest. For the multi-family set, mean values of two SSC measurements were used for further analysis. Five and three field replicates with a single plant per replicate were evaluated for diversity and multi-family trials, respectively. Raw data of all replicates were used as input values for Best Linear Unbiased Estimate (BLUE). BLUE values adjusted for year effects were computed and used for GWAS analysis. GWAS with the BLINK model [43] including the top 5 principal components (PCs) and a kinship matrix were implemented using GAPIT3 software [44]. Phenotypic variances explained by each QTL were estimated as the difference of R^2^ values between the mixed linear model with and without the leading marker. Haploview 4.2 software [45] was used for haploblock identification and visualization using default parameters, except any pairwise comparison of markers was not considered beyond 2 Mb. Haploblocks including top markers were identified for both SSC QTLs. A five-SNP marker block and a 13-SNP marker block were identified for *SSC1* and *SSC2*. Haplotypes representing ≥1% of haplotype diversity at each locus were determined and their effects were estimated for SSC and yield.

### Sugar metabolite profiling

Thirteen metabolites in the starch and sucrose metabolism pathway were evaluated in leaves and in fruit at white and red stages for 23 genotypes. Freeze-dried samples were stored at −80°C. Four technical replicates were evaluated. Sample preparation and LC–MS/MS were conducted according to previous studies [46–48]. Briefly, 10 mg of dried powder were treated with 0.04 ml of IS solution (200 ppb). After extraction with water and centrifugation, supernatant was diluted using acetonitrile (1:1) and filtered through 0.22-μm nylon filter. 4 ul of supernatant was used for LC–MS/MS injection. Authentic standards of sugar metabolites (fructose 6-phosphate, fructose, trehalose 6-phosphate, sucrose, trehalose, glucose, a-D-glucose 1-phosphate, maltose, isomaltose, sucrose 6-phosphate, glucose 6-phosphate, a-D-glucose 1,6-bisphosphate, UDP-glucose, and ADP glucose) were purchased from Sigma-Aldrich (St. Louis, MO, USA). Internal standards (IS) including glucose-13C_6_ and sucrose-^13^C_12_ were purchased from Toronto Research Chemicals (Toronto, ON, Canada). The experimental conditions of LC–MS/MS analyses were similar to previous study [47] except that an Agilent Poroshell 120 HILIC-Z (2.0 × 150 mm, particle size 2.7 μm) column was used for analytes separation and a gradient elution of 10 mM ammonium acetate (pH 9.0) with 0.25 mM methylphosphonic acid in water (eluent A) and 10 mM ammonium acetate (pH 9.0) in water/acetonitrile (10/90, v/v) (eluent B) was performed.

### Expression quantitative locus mapping

An eQTL map was developed in a previous study [49]. Briefly, the total transcriptome from ripe strawberry fruit was sequenced for 196 individuals. Linear mixed models (LMM) implemented in GEMMA were used for GWAS [50], provided with genotyping data of 50 K FanaSNP array and gene expression results. The lead markers in *SSC1* and *SSC2* loci were used to identify co-segregating cis-eQTL for genes underlying those regions. Gene trees were built with maximum likelihood model implemented in RAxML [51].

## Supplementary Material

Web_Material_uhad271

## Data Availability

All raw data is provided in the supplementary files.
