# Supplementary material for: Strawberry soluble solids QTL with inverse effects on yield
Source: Hortic Res. 2023 Dec 21;11(2):uhad271. doi: 10.1093/hr/uhad271 (PMC10873791; doi:10.1093/hr/uhad271)
Supplement: Web_Material_uhad271 [file web_material_uhad271.zip › SupplementaryFigures_09122023.pdf]

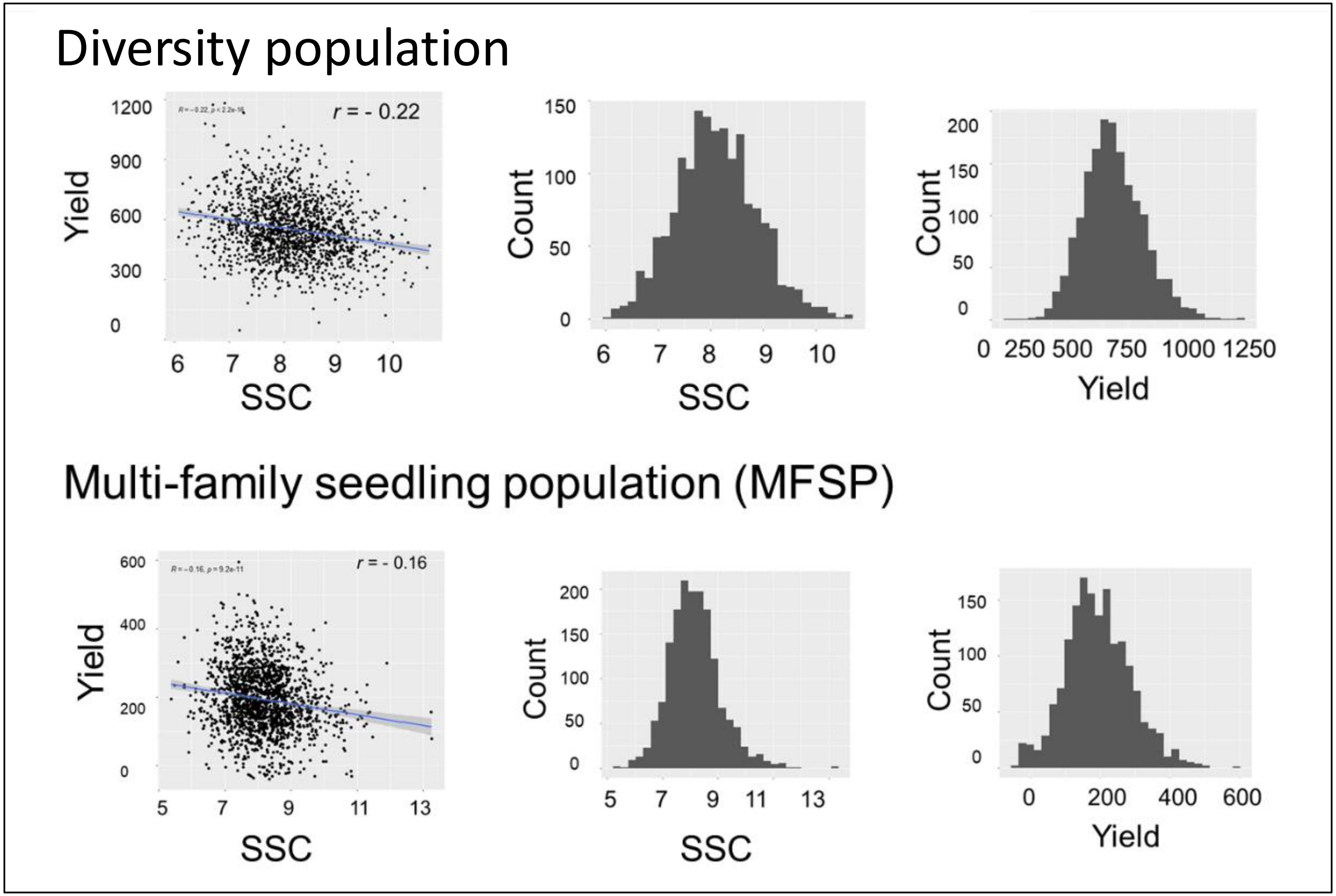

Figure S1. From left to right, dot plot between yield and soluble solids content and the best-fit linear model (the Pearson's correlations are annotated on the top right corner). Histograms of distribution of SSC (middle) and yield (right).

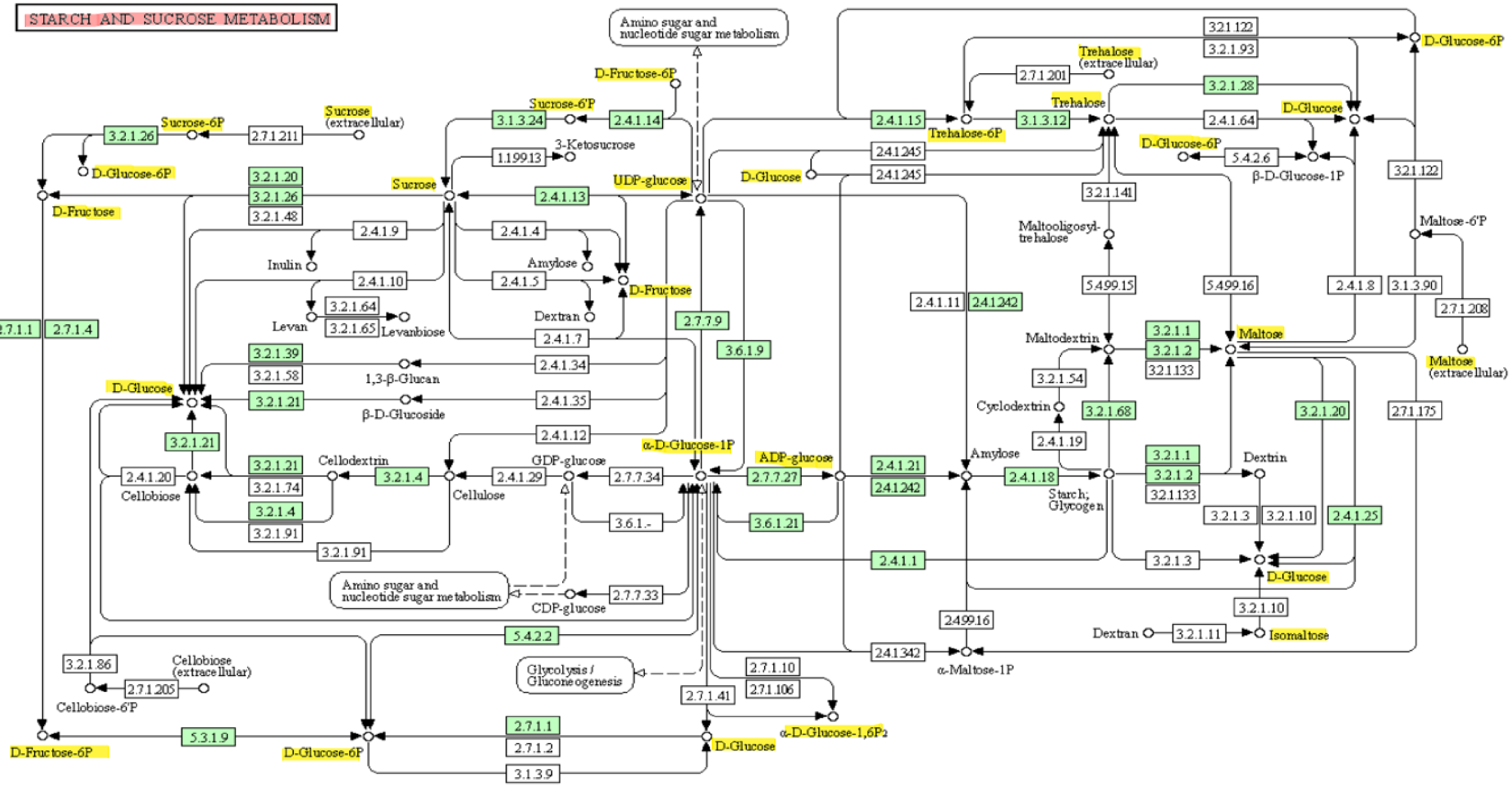

Figure S2. Metabolites in the starch and sucrose metabolism pathway. The sugar metabolites identified and quantified in our work are highlighted in yellow.

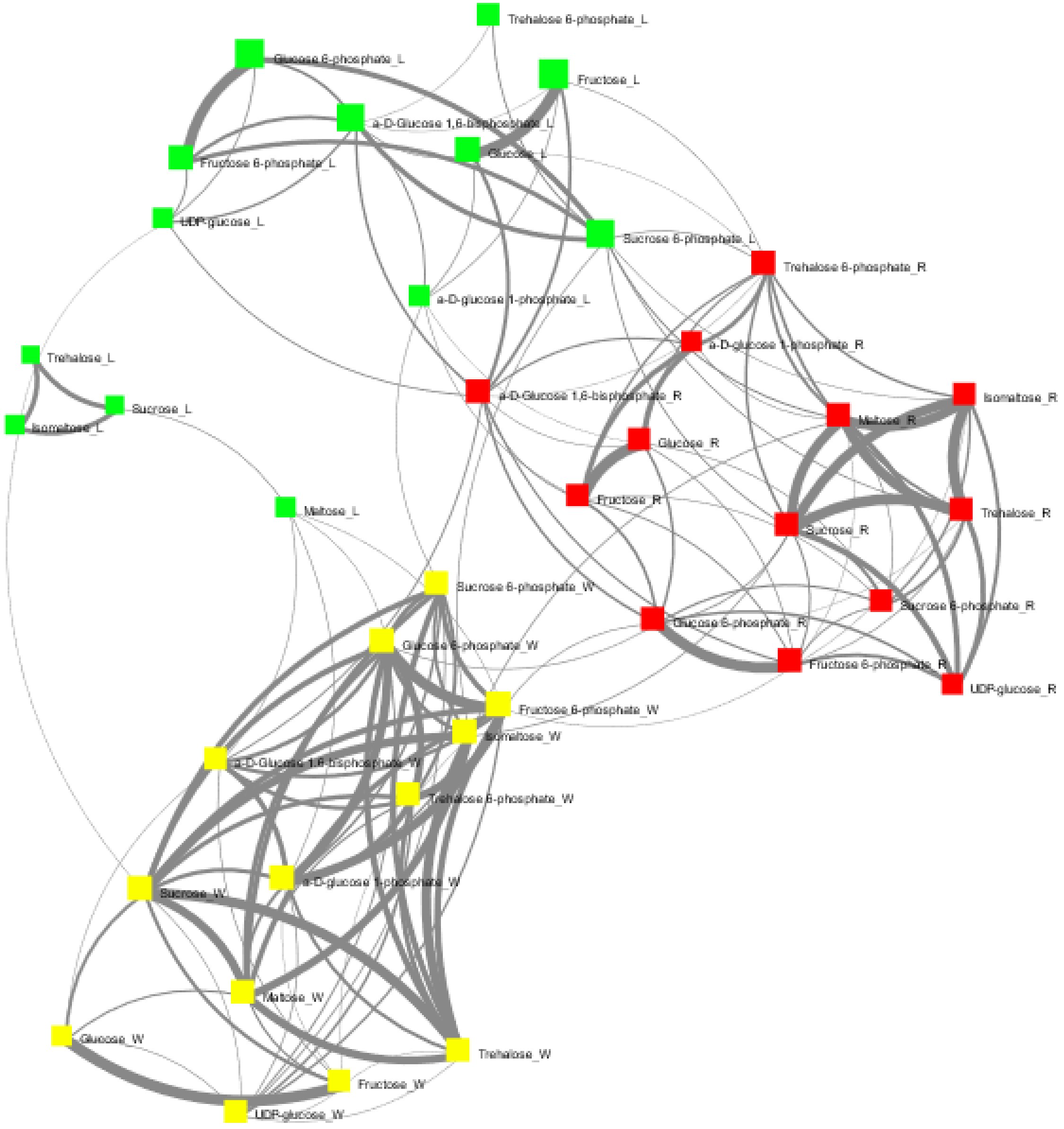

Figure S3. Network analysis of sugar metabolites across three tissue types (green: leaf, red: red fruit, and yellow: white fruit). The edges are proportional to the Pearson's correlations between two nodes.

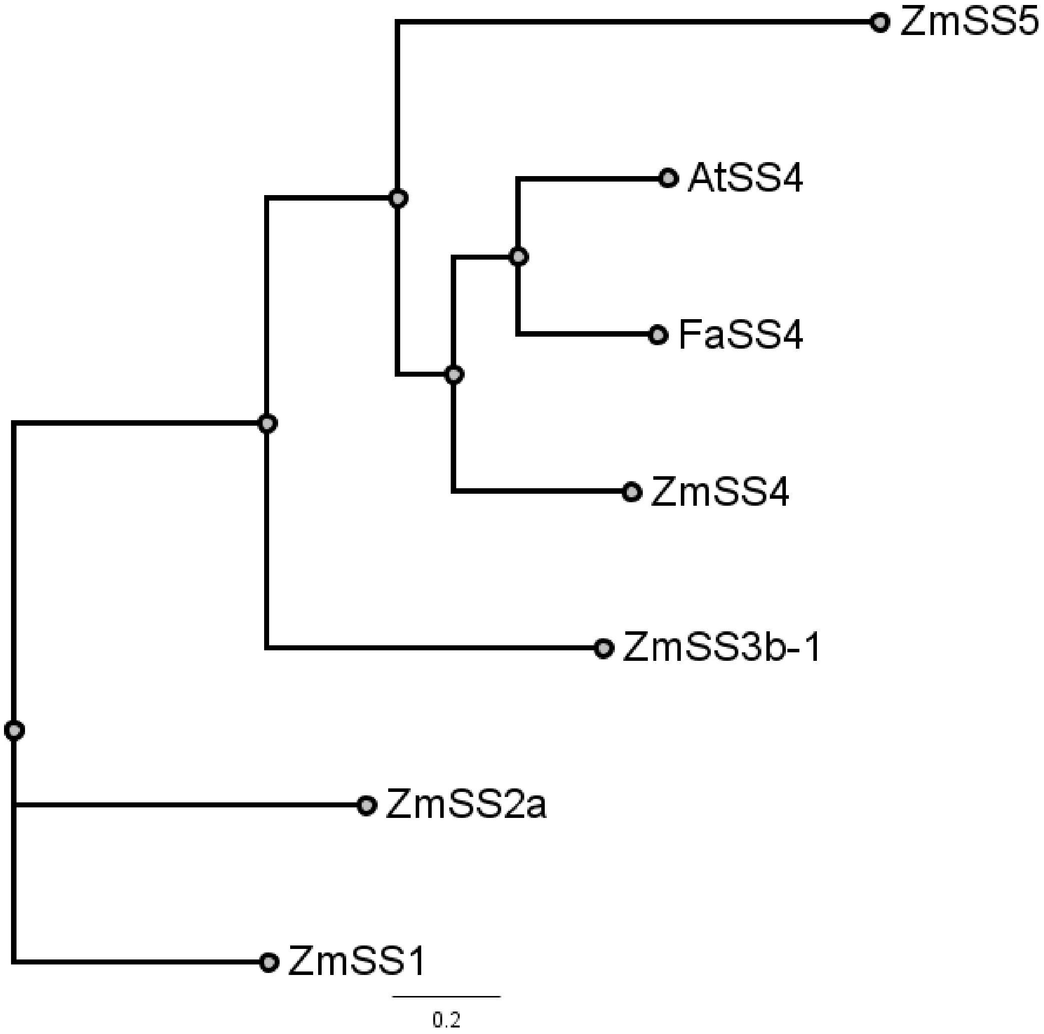

Figure S4. ML tree of starch synthase genes. FaSS4 stands for FxaC_10g00830 within the SSC1 locus. NCBI accession numbers are AtSS4 (NM_117934.4), ZmSS1 (NM_001111422.1), ZmSS3b-1 (NM_001112545.2), ZmSS4(NM_001130118.1), ZmSS5 (NM_001130131.1), and ZmSS2a (NM_001279972.2).

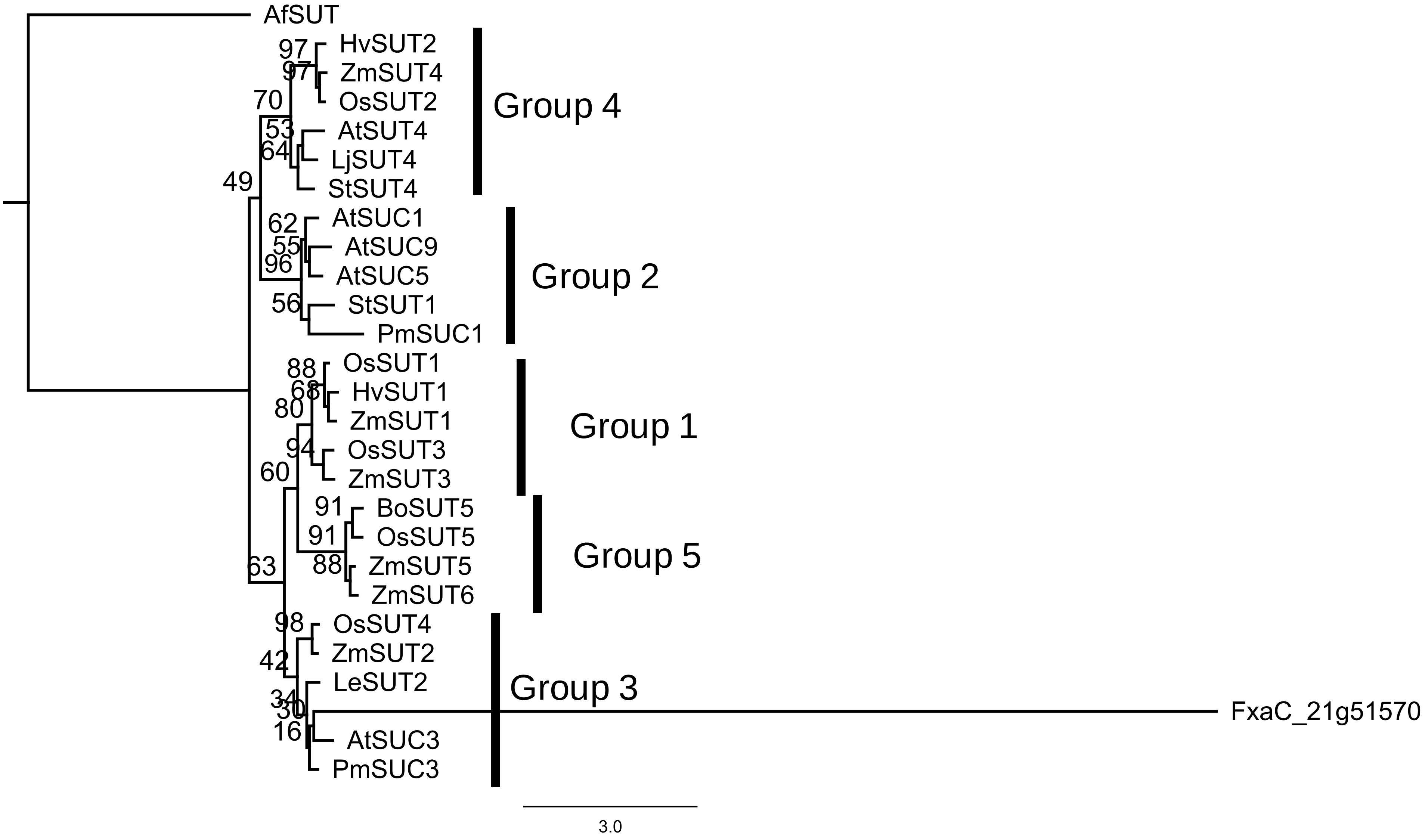

Figure S5. ML tree of sucrose transporters. Branches are labeled with bootstrap values of 100 repeats. SUT-like sequence from Aspergillus fumigatus was used as an outgroup to root the tree. NCBI accessions are OsSUT1 (AAF90181), ZmSUT1 (BAA83501), HvSUT1 (CAB75882), OsSUT2 (BAC67163), ZmSUT2 (AAS91375), HvSUT2(CAB75881), OsSUT3 (BAB68368), ZmSUT3 (ACF86653), OsSUT4 (BAC67164), ZmSUT4 (AAT51689), OsSUT5 (BAC67165), ZmSUT5 (ACF85284), BoSUT5 (AAY43226), ZmSUT6 (ACF85673), AtSUC1 (At1g71880), AtSUC3 (At2g02860), AtSUT4 (At1g09960), AtSUC5 (At1g71890), AtSUC9 (At5g06170), LeSUT2 (AAG12987), LjSUT4 (CAD61275), PmSUC1 (CAI59556), PmSUC3 (CAD58887), StSUT1 (CAA48915), StSUT4 (AAG25923) and AfSUT (EAL92728).
